# Establishment and Internal Validation of a Prognostic Score for Post-hepatectomy Liver Failure Based on Functional Liver Parameters Estimated via TC-99m GSA

**DOI:** 10.7759/cureus.42297

**Published:** 2023-07-22

**Authors:** Meidai Kasai, Sangkil Ha-Kawa, Tsukasa Aihara, Shinichi Ikuta, Takayoshi Nakajima, Naoki Yamanaka

**Affiliations:** 1 Department of Surgery, Meiwa Hospital, Hyogo, JPN; 2 Department of Radiology, Meiwa Hospital, Hyogo, JPN

**Keywords:** the 99mtc-galactosyl human serum albumin, bootstrapped validation, multivariate analysis, gsarmax, prognostic score, tc-99m gsa, post-hepatectomy liver failure

## Abstract

Background

The 99mTc-galactosyl human serum albumin (Tc-99m GSA) scintigraphy evaluates the future remnant liver function, which is an important prognostic factor for post-hepatectomy liver failure (PHLF). This study aimed to establish a new prognostic score for PHLF, including the functional liver parameters evaluated by Tc-99m GSA scintigraphy.

Materials and methods

This study reviewed a single-center, retrospective 368-patient database of those who underwent open and laparoscopic hepatectomy in Meiwa Hospital from January 2016 to October 2021. Moreover, 102 patients who underwent Tc-99m GSA scintigraphy following hepatectomy were analyzed. The index of blood clearance of the tracer was calculated from the uptake ratio of heart at 15 minutes to that at 3 minutes (HH15) and the index of hepatic accumulation was calculated from the uptake ratio of liver to liver plus heart at 15 minutes after the injection (LHL15) were calculated for the general functional parameters. The maximal removal rate of Tc-99m GSA (GSARmax) was also calculated, then the GSARmax of the remnant liver (GSARmax-RL) was estimated as the future remnant liver function depending on the hepatectomy. Multivariate analysis was conducted to identify the PHLF predictor, and then a risk-scoring system was established with the 1,000-times bootstrapped validation.

Results

PHLF (grade ≥ B) was observed in 13 of 102 patients. Multivariate analysis revealed that PHLF was independently predicted by GSARmax-RL (<0.26 mg/min) and LHL15 (<0.89). The risk score was assigned to each item and then classified into four subgroups, with a predicted PHLF of 3.7%, 14.4%, 42.8%, and 76.8%. Receiver operating characteristic (ROC) curve analysis demonstrated good discrimination (adjusted area under the curve (AUC) after bootstrapped validation, 0.779). The ROC curve analysis compared with other prognostic scores showed that the new model had the highest AUC values for accuracy.

Conclusions

The new prognostic score based on Tc-99m GSA scintigraphy could recognize patients with a high risk of progressing to PHLF and be helpful in planning therapeutic strategies.

## Introduction

Impaired remnant liver regeneration following liver surgery is a risk factor for developing posthepatectomy liver failure (PHLF). It occurs in 0.7%-35% of patients who undergo major hepatectomy and is strongly associated with severe morbidity and in-hospital mortality [1]. Various significant predictive factors for PHLF have been identified in previous studies such as liver-associated enzymes, serum bilirubin, prothrombin time, postoperative liver volume calculated, and liver-specific drug stress tests (e.g., indocyanine green retention test at 15 min (ICG-R15)), and prognostic scores have been established [2-4]. Newly developed prognostic scores, e.g., the albumin-bilirubin (ALBI) and albumin-indocyanine green evaluation (ALICE) scores, have been also reported, demonstrating a considerable prognostic PHLF ability [5,6]. These clinical risk scores have guided surgeons to perform appropriate surgical planning to avoid PHLF-related postoperative mortality.

Technetium-99m galactosyl human serum albumin (Tc-99m GSA) scintigraphy is one of the most common nuclear imaging studies in Japan and has been widely used to estimate liver function by counting the asialoglycoprotein receptors on the hepatocellular membrane, which is reportedly associated with liver function [7]. Recently, the development of the Tc-99m GSA technology in combination with single-photon emission computed tomography fused with computed tomography (SPECT/CT) enabled remnant liver function calculation commercially [7]. Nuclear imaging studies are considered to be superior to other conventional tests in terms of PHLF prediction because of their reliability [8].

In our institute, the classical prognostic score invented by Yamanaka et al. in 1986 has been adopted as clinical guidance for safe liver resection [4]. However, this prognostic scoring system, consisting of CT-estimated liver resection rate, indocyanine green (ICG) retention rate, and patient’s age, was introduced at the end of 1980. Thus, the differences in patient background, e.g., age and the methodology in considering liver volume alone as a resection assessment, have rendered this system outdated. Therefore, prediction accuracy in the conventional PHLF risk scores can be improved in the current clinical setting. Thus, this study aimed to establish a new prognostic score for PHLF incorporating future remnant liver function (FRLF) estimated via Tc-99m GSA scintigraphy.

This article was previously posted to the Research Square preprint server on April 6, 2023.

## Materials and methods

This retrospective study was approved by the Meiwa Hospital Ethics Committee. All patients provided written informed consent. A single-center retrospective database of 368 patients with benign and malignant liver diseases who underwent open and laparoscopic hepatectomy in Meiwa Hospital was reviewed from January 2016 to October 2021. Patients had to be examined by preoperative Tc-99m GSA scintigraphy and CT volumetry and undergo the planned anatomical liver resection. Anatomical liver resection consists of monosectionectomies, bisectionectomies, extended hemihepatectomies, and trisectionectomies in this study. Surgical approaches included open, pure laparoscopic, and hybrid methods. Patients who underwent hepatectomy for small-volume liver resection (i.e., partial hepatectomy and monosegmentectomy), those classified as Child-Pugh C, and those with missing data regarding their preoperative liver function (i.e., Tc-99m GSA scintigraphy values) were excluded. The International Study Group of Liver Surgery (ISGLS) provided the classification for posthepatectomy liver failure, and only grades B and C PHLF were considered primary outcomes in this study [9]. Data on age, sex, the American Society of Anesthesiologists (ASA) class, preoperative laboratory data, CT volumetric parameter, Tc-99m GSA scintigraphy parameters, tumor size and diagnosis, resection type, and postoperative courses were extracted. The Clavien-Dindo classification was used to grade postoperative complications [10]. Surgical procedure terminology was described following the Brisbane Nomenclature from the International Hepato-Pancreato-Biliary Association (terminology committees IHPBA 2000 HPB) [11].

Preoperative planning and functional estimation

For conventional liver function tests, the liver biochemical panel and ICG-R15 were evaluated. The volumetric resection rate (RR) was calculated via CT volumetry (Vincent Synapse, Tokyo, Japan). The prognostic score (PS) reported by Yamanaka et al. was adapted for the final surgical planning determination using the following formula: PS = −84.6 + 0.933 × (RR of the volume) + 1.11 × (ICG-R15) + 0.999 × (age) [4]. Surgical planning was judged by the hepatobiliary multidisciplinary team based on PS, which included surgeons, pathologists, oncologists, and radiologists at the center of the current study.

Technetium-99m galactosyl human albumin scintigraphy

Tc-99m GSA scintigraphy was performed for all patients scheduled for nonminor hepatectomy to assess the FRLF. All patients received 3 mg (of 99mTc-GSA 185 MBq) as an IV bolus, and then dynamic abdominal digital images were obtained using a large field of gamma camera view (E.CAM, Canon Medical Systems Co., Otawara, Japan) equipped with low-energy, high-resolution, parallel-hole collimator centered on the liver and the precordium. Finally, SPECT images were acquired 37-38 min after injection. The index of blood clearance of the tracer was calculated from the uptake ratio of heart at 15 minutes to that at 3 minutes (HH15) and the index of hepatic accumulation was calculated from the uptake ratio of liver to liver plus heart at 15 minutes after the injection (LHL15) were calculated for the general functional parameters. The maximal Tc-99m GSA removal rate (GSARmax) of the whole liver was calculated using the approach described by Kown et al. GSARmax also indirectly estimates asialoglycoprotein (ASGPR) activity in hepatocytes by a radiopharmacokinetic model [12,13]. Finally, the regional GSARmax (anterior, posterior, medial, and left lateral sections) was estimated following the region of interest (ROI) divided by anatomical landmarks, e.g., the hepatic vein. Then, the GSARmax of the remnant liver (GSARmax-RL) was calculated as a remnant liver function representative, depending on the planned hepatectomy.

Statistical analyses and model development

The primary objective of our research was to create and validate a model to predict the risk of PHLF. As our study employed a retrospective observational study, we incorporated data from all patients who fit our predefined inclusion and exclusion criteria. Consequently, there was no separate calculation for sample size. For continuous data, the Mann-Whitney U test was applied for univariate analysis. In contrast, categorical data underwent Fisher’s exact test to identify potential correlations with PHLF. Risk factors that showed significance (P < 0.05 in univariate analysis) proceeded to multivariate logistic regression to adjust for confounders. Using the receiver operating characteristic (ROC) curve analysis, continuous variables were assessed and subsequently dichotomized based on the optimal cutoff point for inclusion in the logistic regression models. A backward stepwise approach in multivariate logistic regression was then employed. Drawing from the methodology of the Framingham Heart Study [14], we developed a points system to gauge the risk of PHLF. Through binary logistic regression, we identified the independent risk factors for PHLF, assigning points based on the coefficients of the refined models. Once the scoring system was established, the corresponding risk for each point total was evaluated. It's worth noting that a similar approach has been adopted in hepatobiliary-pancreatic surgical research, particularly as seen in the study by Halls et al. [15].

The performance of the models was evaluated in terms of discrimination and calibration. Discrimination was assessed using the ROC curve. Calibration was assessed using the calibration plot to compare the differences between the predicted and actual incidence rates. The prognostic model was internally validated with a bootstrap method with 1,000 resamples to estimate overfitting and optimism. Optimism was calculated to correct an area under the curve (AUC) for the ROC curve following the methodology described by Steyerberg et al. [16]. Finally, the new prognostic scoring system of the current study was compared with other existing scoring systems, e.g., ALICE score, ALBI score, and Yamanaka’s PS, demonstrating their ROC curves. The statistical significance was set at P < 0.05, and all statistical analyses were performed using R software, version 4.1.3 (http://cran.r-project.org/). The risk score was developed following the transparent reporting of a multivariable prediction model for the individual prognosis or diagnosis methodology [17].

## Results

This study included 102 (26 women and 74 men) patients; 13 of whom developed PHLF grade B. No patient developed PHLF grade C. The study population background is summarized in Table 1.

**Table 1:** Study population background BMI: body mass index; PHLF: posthepatectomy liver failure; NBNC: non-HBV non-HCV; ASA: American Society of Anesthesiologists; CRLM: colorectal liver metastasis; HCC: hepatocellular carcinoma; ICC: intrahepatic cholangiocarcinoma; CCC: cholangiocellular carcinoma; GB: gallbladder; RH: right hepatectomy; exRH: extended right hepatectomy; LH: left hepatectomy; exLH: extended left hepatectomy; RAS: right anterior sectionectomy; RPS: right posterior sectionectomy; LLH: left lateral sectionencomy; LMS: left medial sectionectomy; CH: central hepatectomy

Variables	All patients (n = 102)	Non-PHLF group (n = 89)	PHLF group (n = 13)	P values
Age, years, median (range)	72 (37–88)	72 (38–88)	72 (37–85)	0.904
Sex, male, n (%)	74 (72.3%)	63 (71%)	11 (85%)	0.506
BMI, median (range)	22.61 (15.4–35.6)	22.68 (15.4–33.4)	21.45 (16.4–35.6)	0.281
Etiology				
HBV, n (%)	9 (8.8%)	6 (7%)	3 (23%)	0.087
HCV, n (%)	15 (14.7%)	11 (12%)	4 (31%)	0.097
NBNC, n (%)	78 (76.5%)	72 (81%)	6 (46%)	0.011
Indication				0.932
CRLM, n (%)	25 (24.5%)	23 (26%)	2 (15%)	
HCC, n (%)	45 (43.1%)	35 (40%)	9 (69%)	
ICC, n (%)	14 (13.7%)	12 (14%)	2 (15%)	
Hilar CCC, n (%)	6 (5.9%)	6 (6.7%)	0	
GB cancer, n (%)	2 (2.0%)	2 (2%)	0	
Hemangioma, n (%)	1 (1.0%)	1 (1.1%)	0	
IPNB, n (%)	1 (1.0%)	1 (1.1%)	0	
Other malignancy, n (%)	7	7	0	
Tumor size, cm, median (range)	4.45 (1–25)	4 (1–20)	7 (2–25)	0.120
Number of tumors, median (range)	1 (1–24)	1 (1–24)	1 (1–5)	0.385
ASA classification Ⅱ/Ⅲ/Ⅳ, n	22/79/1	19/69/1	3/10/0	1.00
Repeated hepatectomy, n (%)	9 (8.8%)	7 (8%)	2 (15%)	0.322
Preoperative chemotherapy, n (%)	41 (40.0%)	35 (39%)	6 (46%)	0.764
Surgical approach Open/hybrid/pure lap, n	82/8/12	71/8/10	11/0/2	0.629
Operation				0.238
Trisectionectmy exRH/exLH, n	2/5	1/4	1/1	
Bisectionectmy RH/LH/CH, n	32/21/2	29/19/1	3/2/1	
Monosectionectomy LLH/LMS/RAS/RPS, n	5/5/18/18	5/5/16/14	0/0/2/5	

A significant difference was observed in the non-B-non-C liver status proportion between the non-PHLF and PHLF groups (81% vs. 46%, p = 0.011). No significant differences were observed in conventional biochemical liver panels between the two groups except for prothrombin activity (89% vs. 80%, p = 0.002), the proportion of Child classification B (5% vs. 23%, p = 0.042), ALBI score (−2.57 vs. −2.18, p = 0.047), ALICE score (−2.02 vs. −1.61, p = 0.048;), HH15 (0.59 vs. 0.67, p = 0.014), LHL15 (0.92 vs. 0.88, p = 0.004), and GSARmax-RL (0.39 mg/dl vs. 0.26 mg/dl, p = 0.009) between both groups, respectively, in Table 2.

**Table 2:** Preoperative laboratory data and estimated liver function AST: aspartate transaminase; ALT: alanine aminotransferase; ICG15R: indocyanine green retention test at 15 min; ALBI: albumin-bilirubin; ALICE: albumin-Indocyanine green evaluation; PS: prognostic score reported by Yamanaka in 1986 [4]; RL: remnant liver; HH15: the uptake ratio of heart at 15 minutes to that at 3 minutes; LHL15: the uptake ratio of liver to liver plus heart at 15 minutes; GSARmax: the maximal Technetium-99m galactosyl human serum albumin removal rate; BSA: body surface area

Variables	All patients (n = 102)	Non-PHLF group (n = 89)	PHLF group (n = 13)	P values
Total bilirubin, median (range), mg/dl	0.7 (0.2–4.5)	0.7 (0.2–4.5)	0.8 (0.3–2.9)	0.442
Serum albumin, median (range), g/L	3.9 (2.5–4.6)	3.9 (2.5–4.6)	3.4 (2.5–4.1)	0.056
AST, median (range), U/L	36 (11–157)	35 (11–157)	48 (14–143)	0.126
ALT, median (range), U/L	27 (7–170)	27 (7–161)	37 (9–170)	0.710
Prothrombin activity, median (range), % (unit)	104 (60–124)	89 (60–124)	80 (63–104)	0.002
Hemoglobin, median (range), g/L	12.75(8.2–17)	12.7 (8.2–17)	13.3 (9.1–16.2)	0.356
Platelet count, median (range), /uL	18.2(6.3–55.1)	18.2 (6.3–55.1)	21.8 (11.1–33.1)	0.637
ICG15R, median (range), % (unit)	16.65(1.21–51.8)	16.4 (1.21–51.8)	19.3 (4–32.9)	0.212
Child classification (A/B), n	95 /7	85/4	10/3	0.042
Liver damage Classification (A/B), n	81/21	73/16	8/5	0.135
ALBI score, median (range)	−2.54 (−3.21 to –1.14)	−2.57 (−3.21 to –1.21)	−2.18 (−3.02 to –1.14)	0.047
ALBI grade: 1/2a/2b/3, n	47/21/31/3	43/20/24/2	4/1/7/1	0.104
ALICE score, median (range)	1.98(−2.8 to –0.83)	−2.02 (−2.8 to –0.88)	−1.61 (−2.4 to –0.83)	0.048
ALICE grade: 1/2/3, n	30/62/10	29/52/8	1/10/2	0.120
Yamanaka’s PS [4], median (range)	37.05(−9.8–82.7)	36.3 (−9.8–82.7)	43.2 (16.5–63.4)	0.112
HH15, median (range)	0.59(0.4–0.85)	0.59 (0.4–0.77)	0.67 (0.51–0.85)	0.014
LHL15, median (range)	0.92 (0.77–1)	0.92 (0.77–1)	0.88(0.82–0.94)	0.004
GSARmax, median (range), mg/min	0.57(0.19–1.08)	0.58(0.19–1.08)	0.55 (0.21–0.76)	0.079
GSARmax-RL, median (range), mg/min	0.37(0.12–0.77)	0.39(0.12–0.77)	0.26 (0.14–0.52)	0.009
Resection rate of GSARmax, median (range)	0.3(0.09–0.67)	0.29(0.09–0.67)	0.32 (0.13–0.66)	0.748
Remnant liver volume, median (range), ml	765(344–1,703)	763(344–1,703)	796 (463–1,310)	0.707
Resection rate of liver volumes, median (range), ml	0.34(0.08–0.65)	0.34(0.09–0.65)	0.37 (0.08–0.62)	0.316
Remnant liver volumes per weight, median (range), ml/kg	1.3(0.62–2.56)	1.31(0.62–2.56)	1.2 (0.73–2.16)	0.718
Remnant liver volumes per BSA, median (range), ml/m2	460.2(221.19–886.22)	477.98(221.19–886.22)	451.84(269.8–732.18)	0.688

In the perioperative parameters, as summarized in Table 3, estimated blood loss was significantly lower in the non-PHLF group (820 ml vs. 1,700 ml, p = 0.017), postoperative length of stay was also shorter (19 days vs. 29 days, p < 0.001), and postoperative bile leakage was less likely (15% vs. 38%, p = 0.040) compared with the PHLF group.

**Table 3:** Clinical outcomes CD: Clavien–Dindo; SSI: surgical site infection; DGE: delayed gastric emptying; PV: portal vein; PHLF: posthepatectomy liver failure

Variables	All patients (n = 102)	Non-PHLF group (n = 89)	PHLF group (n = 13)	P values
Operative time, median (range), min	413 (173–877)	414 (173–730)	394 (287–877)	0.165
Blood loss, median (range), ml	920 (10–9,330)	820 (10–9,330)	1,700(620–9,010)	0.017
Blood transfusion rate, n (%)	34 (33.3%)	28 (31%)	6 (46%)	0.350
Pringle maneuverer, median (range), min	35.5 (0–189)	35 (0–189)	37 (0–169)	0.968
Postoperative length of stay, median (range), days	20 (6–130)	19 (6–71)	29 (20–130)	<0.001
Morbidity CD grade Ⅰ–Ⅱ/Ⅲa–Ⅲb/Ⅳ, n	67/18/1	60/13/0	7/5/1	0.001
Description of morbidity: any CD grade				
Bile leakage, n (%)	18 (17.7%)	12 (15%)	5 (38%)	0.0398
SSI, n (%)	25 (24.5%)	21 (24%)	4 (31%)	0.730
Pneumonia, n (%)	7 (6.9%)	5 (6%)	2 (15%)	0.218
Cholangitis, n (%)	2 (2.0%)	2 (2%)	0 (0%)	1.0
Ascites, n (%)	2 (2.0%)	0	2 (15%)	0.015
PV thrombosis, n (%)	2 (2.0%)	2 (2%)	0 (0%)	1.0
DGE, n (%)	3 (2.9%)	3 (3%)	0 (0%)	1.0
Postoperative bleeding, n (%)	2 (2.0%)	1 (1%)	1 (8%)	1.0
PHLF grade A/B/C, n	52/13/0	52/0/0	0/13/0	<0.001
Mortality in 30 days for any reason, n	0	0	0	1.0
Mortality in 90 days for any reason, n	0	0	0	1.0

The establishment of a clinical prognostic score with multivariate analysis

Upon univariate analysis, eight preoperative variables (non-B-non-C liver status, prothrombin activity (PT), Child classification B, ALBI score, ALICE score, HH15, LHL15, and GSARmax-RL) were possible PHLF predictive factors. Multivariate analysis identified two significant factors in the final model, with the best cutoff values according to the ROC analysis: GSARmax-RL (<0.26 mg/min; odds ratio (OR) = 15.8, 95% confidence interval (95% CI) = 3.7-77.4, P < 0.001), LHL15 (<0.89; OR = 6.79, 95% CI = 1.6-31.7, P = 0.011). Based on the coefficient, risk points were assigned to each item to build the clinical PS, and the total score ranged from 0 to 3 points (GSARmax-RL, 2 points; LHL15, 1 point; Table 4).

**Table 4:** Multivariate analysis result of significant factors identified by univariate analysis OR: odds ratio; CI: confidence interval; NBNC: non-HBV, non-HCV; ALBI: albumin-bilirubin; ALICE: albumin-indocyanine green evaluation; HH15: the uptake ratio of heart at 15 minutes to that at 3 minutes; LHL15: the uptake ratio of liver to liver plus heart at 15 minutes; GSARmax: the maximal Technetium-99m galactosyl human serum albumin removal rate; RL: remnant liver

Variable	Coefficient	OR	95% CI	P value	Newly Assigned Predicted Score
NBNC status	−0.97	0.38	2.19–0.07	0.26	–
Prothrombin activity < 85%	1.47	4.36	23.0–0.97	0.06	–
Child classification B	0.71	2.04	26.09–0.16	0.57	–
ALBI score ≥ -2.18	1.38	3.98	44.88–0.34	0.26	–
ALICE score ≥ -1.61	−1.03	0.36	3.69–0.03	0.39	–
HH15 < 0.67	−0.05	0.96	7.17–0.16	0.96	–
LHL15 < 0.89	1.92	6.79	31.72–1.56	0.01	1
GSARmax-RL < 0.26 (mg/min)	2.76	15.8	77.44–3.65	<0.01	2

**Figure 1:**
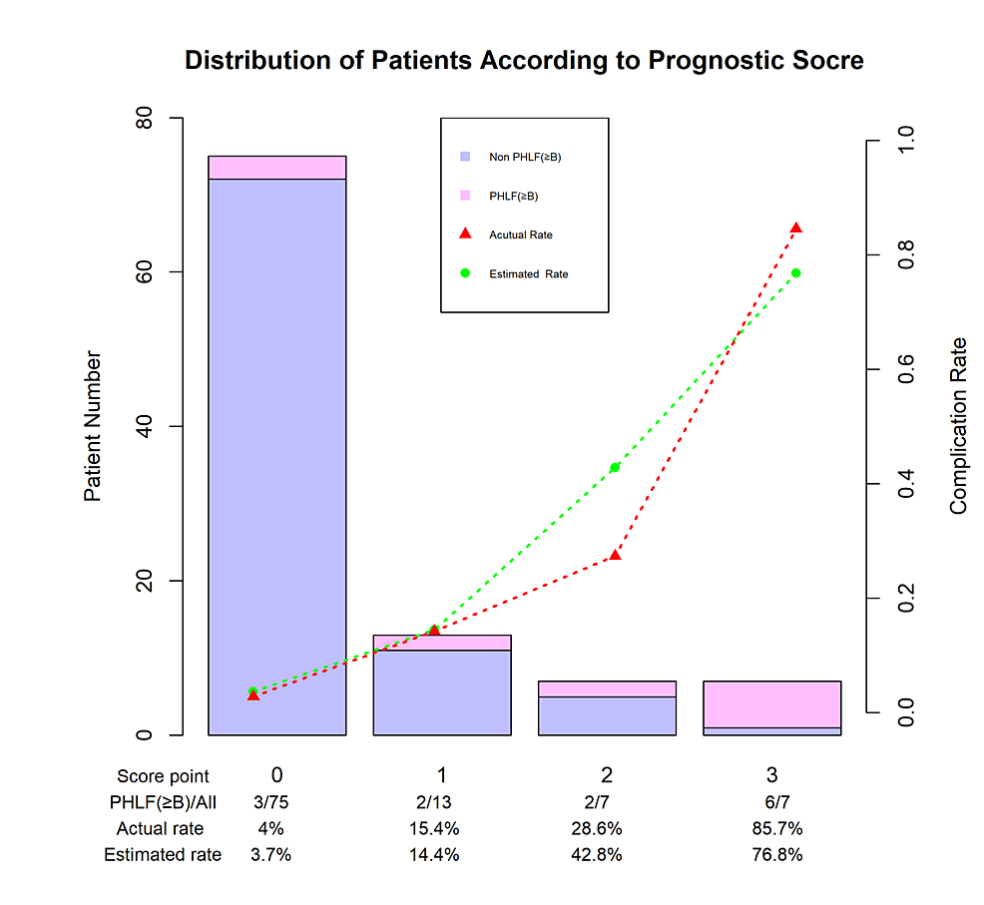
Predicted PHLF risks and observed proportions according to prognostic score. PHLF posthepatectomy liver failure;

Furthermore, the patients were grouped into four subgroups with a predicted PHLF of 3.7%, 14.4%, 42.8%, and 76.8% (Figure 1) wherein the actual and estimated PHLF incidence was compared.

**Figure 2:**
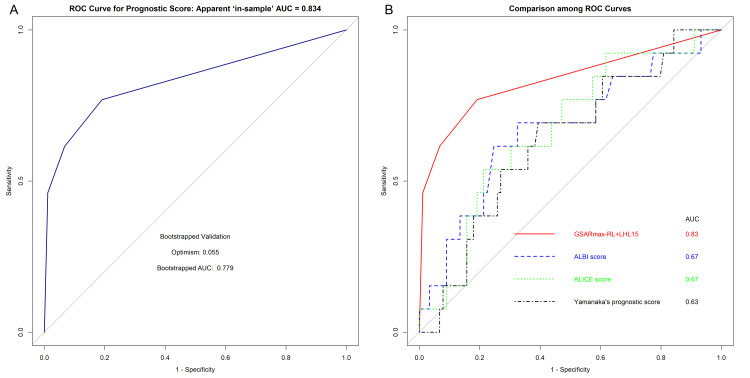
ROC curve 2a. The new prognostic ROC curve with a bootstrapped AUC value. Bootstrapped validation demonstrated optimism (0.055) and adjusted AUC (0.779). 2b. Multiple comparisons among the ROC curves of the new prognostic score, ALBI score, ALICE score, and PS by Yamanaka [4]. ROC: receiver operating characteristic; AUC: area under the curve; GSARmax: the maximal Technetium-99m galactosyl human serum albumin removal rate; LHL15: the uptake ratio of liver to liver plus heart at 15 minutes; RL: remnant liver; ALBI: albumin-bilirubin; ALICE: albumin-indocyanine green evaluation; PS: prognostic score reported by Yamanaka in 1986 [4]

 The ROC curve demonstrated good discrimination with an AUC of 0.834 (95% CI, 0.6967-0.9714; Figure 2a). Internal validation of the best-fit model with 1,000 bootstrap resamples showed that the average optimism was 0.055 with the adjusted AUC of 0.779 showing an acceptable discrimination ability. Multiple comparisons with other prognostic scores (i.e., ALICE score, ALBI score, PS by Yamanaka) showed that the new model in the current study had the highest AUC values (Figure 2b).

**Figure 3:**
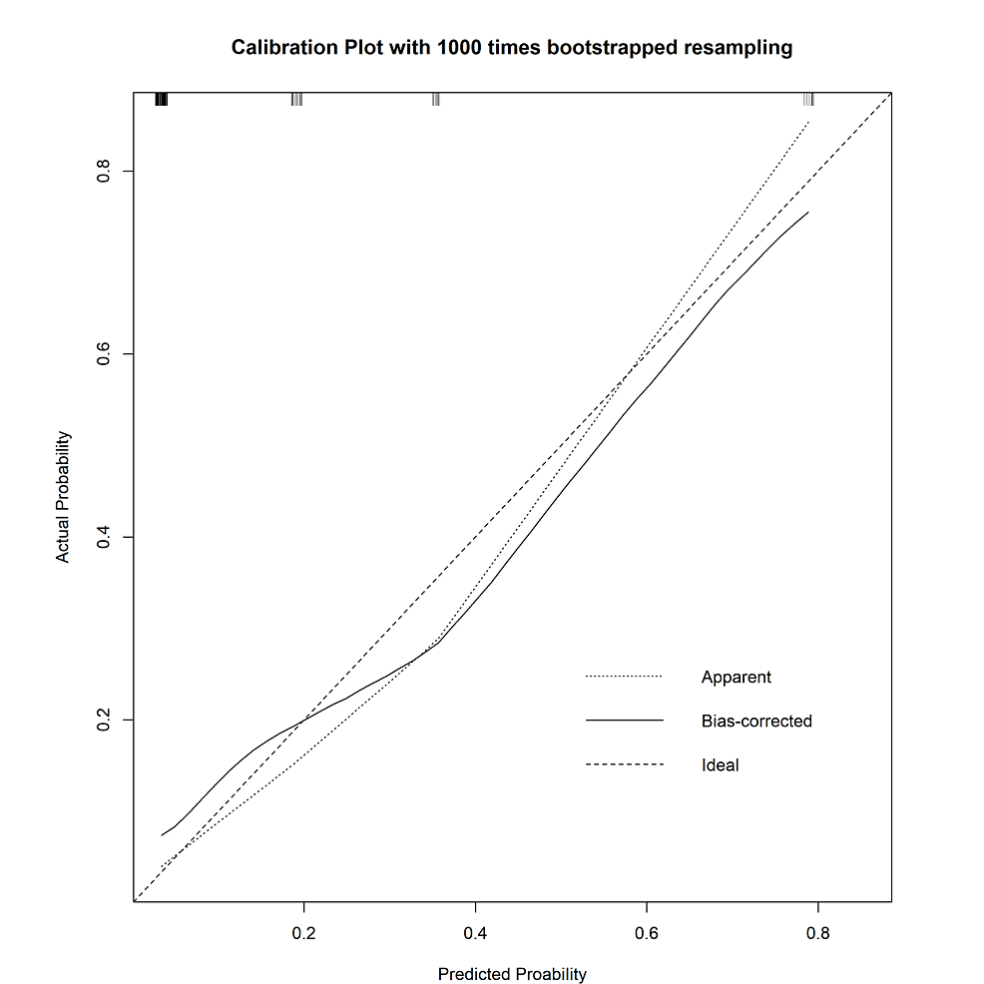
Calibration curves constructed by the bootstrap approach A good correlation between observation and prediction is demonstrated, but the apparent curve and bias-corrected curve slightly deviated from the references.

The calibration curve was close to the 45° ideal line (Figure 3), demonstrating good agreement between the predicted and actual PHLF incidence and suggesting only a small degree of bias from overfitting in the best-fit model.

## Discussion

Tc-99m GSA was developed by Vera et al. in 1985 and has been gaining popularity worldwide as a nuclear imaging test for the functional assessment of not only the entire liver but also the regional liver, combined with SPECT/CT [17-19]. This inversional test is highly reproducible, in contrast with the ICG test, because it specifically binds to asialoglycoprotein receptors and its biochemical processes are completely independent of hepatic blood flow and serum bilirubin [7]. Several parameters related to Tc-99m GSA have been currently reported, e.g., HH15, LHL15, liver uptake density (LUD), LU15, and GSARmax [20] as shown in Table 5 [12,13,20-25].

**Table 5:** Commonly used parameters from dynamic parameters of GSA scintigraphy LHL15: the uptake ratio of liver to liver plus heart at 15 minutes; 99mTc-GSA: technetium-99m galactosyl human albumin scintigraphy; HH15: the uptake ratio of heart at 15 minutes to that at 3 minutes; LU15: ratio of uptake by the liver at 15 min to the injected dose of 99mTc-GSA; LUD: liver uptake density; GSARmax: the maximal technetium-99m galactosyl human serum albumin removal rate; RL: remnant liver

Abbreviation	Parameter	Description	Reported cut-off values for predicted liver dysfunction
LHL15	Hepatic uptake ratio of 99mTc-GSA	Liver counts at 15 min (L15) divided by heart counts (H15) plus L15	LHL15 < 0.9 [21]
HH15	Blood clearance ratio	Heart counts at 15 min (H15) divided by heart counts at 3 min (H3)	HH15 > 0.55 [22]
LU15	Liver uptake	Cumulative liver uptake 15–16 min after injection from liver time–activity curve (L(t))	LU15 < 13 [23]
LUD, %	Liver uptake ratio	Liver uptake ratio divided by liver functional volume	LUD > 0.04% [24]
GSARmax, mg/min	Maximal removal rate of 99mTc-GSA	Calculated with the kinetic model of Ha-Kawa et al. [25]	GSARmax < 0.35 mg/min [13], GSARmax-RL < 0.15 mg/min [14]

Concerning GSARmax, Kwon et al. first reported its efficacy in 1997, suggesting their original five-compartment models and an approximate formula to mathematically calculate it [13]. They reported preoperative that GSARmax (<0.35 mg/min) and GSARmax-RL (<0.15 mg/min) were found to be significant independent risk factors for postoperative PHLF mortality following major hepatectomy in subsequent studies [14]. The cutoff GSARmax-RL value in the current study was 0.26 mg/min, which is a higher value than the previous one. This difference possibly reflects the difference in the primary outcome between the current study and others. The primary outcome of the current study was PHLF, whereas the primary outcome of other studies was PHLF-associated mortality.

Whether the remnant liver function is accurately estimated by the future remnant of liver volumes (FRLV) assessed via CT volumetry has been debated. CT volumetry‑based function indirectly estimated capacity by assuming that liver function is homogenously distributed across all liver regions. This is not a universal concept, especially for patients with underlying parenchymal liver diseases [8]. Thus, the direct liver metabolic ability measurement by nuclear imaging studies, e.g., Tc-99m GSA, is more suitable to evaluate the true functional liver remnant capacity. To support this idea, recent studies from Blüthner et al. demonstrated that the FRLF assessed via the nuclear imaging test is superior to FRLV in terms of postoperative morbidity prediction, e.g., PHLF, postoperative ascites, hemorrhage, and poor wound healing in cirrhotic and noncirrhotic patients [26,27]. The current study showed that CT volumetric parameters were not associated with PHLF as stated above, whereas the scintigraphy parameters were significant predictive factors. No published prognostic scores exist for PHLF based on FRLF estimated by Tc-99m GSA despite the promising published nuclear imaging studies data in the assessment of surgical respectability and eligibility. The probable reason is that no clear guidelines exist for surgical management referring to nuclear imaging studies. According to the systematic review from Espersen et al. in 2021, 25 studies demonstrated the relationship between nuclear imaging studies and postoperative morbidity, including PHLF, and considerable heterogeneity was observed across the studies in the methodology [8]. With regards to nuclear tracers, 12 of 25 studies used Tc-99m GSA; however, 11 studies used Tc‑mebrofenin, and two studies used positron emission-based tracers.

Compared to the classic PS invented by Yamanaka in 1986, which was constructed by volumetric RR (ICG15R) and age, this classical prediction system was not a significant factor in the analysis of the current study. Considerably high-risk patients were excluded from the preoperative assessment because the patients in the current study were all given classic PS. In addition, age is no longer considered a crucial factor in developing liver failure due to surgical technique advancement and medical care. In the present study, an important step was made toward qualifying and predicting the PHLF risk with preoperative scintigraphy functional parameters. The risk-scoring system of the current study can predict the PHLF incidence based on only two parameters: GSARmax-RL and LHL15. Furthermore, this system can be used for patients in the clinical setting, showing good discrimination with risk points of 0, 1, 2, and 3 having risk rates of 3.7%, 14.4%, 42.8%, and 76.8% of PHLF incidence rate, respectively. It's clear that patients with a score of 3 are at the highest risk of posthepatectomy liver failure (PHLF). For these patients, considering alternative therapeutic approaches rather than liver resection, or the provision of more intensive care measures, such as additional prophylactic interventions, comprehensive preoperative nutrition, and respiratory therapy, may be beneficial. According to Lei et al., the preoperative phase offers a valuable opportunity for multidisciplinary teams to optimize the health status of the surgical candidate, which could potentially improve perioperative outcomes [28]. Strategies such as prehabilitation could play a crucial role in preparing these patients for surgery, enhancing their resilience and ultimately their recovery. Moreover, transarterial chemoembolization (TACE) combined with radiofrequency ablation (RFA) may also provide a viable alternative to surgical resection (SR) for HCC patients. According to Gui et al., this combination offers comparable oncologic outcomes to surgery in patients with HCC, with the added benefit of lower associated morbidity [29].

Moreover, recent technological advancements in the surgical field have been swift and ongoing. The integration of technologies such as artificial intelligence (AI) with the Internet of Things (IoT) is transforming surgical management [30]. These innovative approaches hold great promise for creating more precise risk scores in the future, potentially improving patient outcomes.

Our study has several limitations. First, its retrospective nature may have introduced unmeasured confounding factors associated with PHLF, inherent to any observational study. Second, although our study included a variety of tumor types, with different clinical courses, our analysis did not find the indication for surgery to be a significant factor affecting outcomes. Third, as aforementioned, nuclear imaging study methodologies differ among countries significantly. Finally, the sample size of the current study was not large enough to be validated in external cohorts, although the scoring system was validated by the 1,000-times bootstrapped resampling technique. Thus, future studies should include a larger sample size of patients.

## Conclusions

This research is vital given the importance of mitigating PHLF, a significant concern in medical practice. Our participant group consisted of patients who underwent hepatectomy for various indications. We devised and validated the first-ever predictive scoring system for PHLF, as defined by the ISGLS, utilizing Tc-99m GSA scintigraphy. Our research concluded that the PHLF risk is primarily stratified by two factors: GSARmax of the remnant liver and LHL15. This novel scoring system enables the identification of high-risk patients, potentially influencing therapeutic strategies in liver surgery, and has broad relevance across medical practice. Patients at high risk for posthepatectomy liver failure must be candidates for limited liver resection or other therapeutic approaches, e.g., chemotherapy, radiotherapy, and transarterial chemoembolization according to the patient‘s clinical background. This work thus underscores the necessity of tailoring treatment strategies to individual patient risk profiles to prevent PHLF-associated mortality.
